# Measurements of Functional Responses in Human Primary Lung Cells as a Basis for Personalized Therapy for Cystic Fibrosis

**DOI:** 10.1016/j.ebiom.2014.12.005

**Published:** 2014-12-17

**Authors:** Nikhil T. Awatade, Inna Uliyakina, Carlos M. Farinha, Luka A. Clarke, Karina Mendes, Amparo Solé, Juan Pastor, Maria Margarida Ramos, Margarida D. Amaral

**Affiliations:** a University of Lisboa, Faculty of Sciences, BioFIG-Center for Biodiversity, Functional and Integrative Genomics, Campo Grande, C8 bdg, 1749-016 Lisboa, Portugal; b Adult Cystic Fibrosis Unit, University Hospital la Fe, Boulevar Sur, 46026 Valencia, Spain; c Thoracic Surgery Service, University Hospital la Fe, Av. Campanar 21, 46009 Valencia, Spain

**Keywords:** CF, cystic fibrosis, CFTR, cystic fibrosis transmembrane conductance regulator, (ΔIeq-sc), equivalent short-circuit currents, ENaC, epithelial Na^+^ channel, Fsk, forskolin, Gen, Genistein, HBE (cells), human bronchial epithelial cells, SEM, standard error of the mean, TEER, transepithelial electrical resistance, Vte, transepithelial voltage, Rte, transepithelial resistance., Innovative treatments, Mutation-specific therapies, Personalized medicine, Rare diseases

## Abstract

**Background:**

The best investigational drug to treat cystic fibrosis (CF) patients with the most common CF-causing mutation (F508del) is VX-809 (lumacaftor) which recently succeeded in Phase III clinical trial in combination with ivacaftor. This corrector rescues F508del-CFTR from its abnormal intracellular localization to the cell surface, a traffic defect shared by all Class II CFTR mutants. Our goal here is to test the efficacy of lumacaftor in other Class II mutants in primary human bronchial epithelial (HBE) cells derived from CF patients.

**Methods:**

The effect of lumacaftor was investigated in primary HBE cells from non-CF and CF patients with F508del/F508del, A561E/A561E, N1303K/G542X, F508del/G542X and F508del/Y1092X genotypes by measurements of Forskolin plus Genistein-inducible equivalent short-circuit current (I_eq-SC-Fsk + Gen_) in perfused open-circuit Ussing chambers. Efficacy of corrector C18 was also assessed on A561E/A561E and F508del/F508del cells.

**Results:**

Our data indicate that A561E (when present in both alleles) responds positively to lumacaftor treatment at equivalent efficacy of F508del in primary HBE cells. Similarly, lumacaftor has a positive impact on Y1092X, but not on N1303K. Our data also show that cells with only one copy of F508del-CFTR respond less to VX-809. Moreover, there is great variability in lumacaftor responses among F508del-homozygous cells from different donors. Compound C18 failed to rescue A561E-CFTR but not in F508del-CFTR, thus plausibly it has a different mechanism of action distinct from lumacaftor.

**Conclusions:**

CF patients with A561E (and likely also those with Y1029X) can potentially benefit from lumacaftor. Moreover, the methodology used here exemplifies how ex vivo approaches may apply personalized therapies to CF and possibly other respiratory diseases.

## Highlights

• A561E and Y1092X CFTR mutations (but not N1303K) respond positively to lumacaftor treatment.
• One copy of F508del-CFTR responds less to lumacaftor than 2 copies and F508del-responses vary greatly.
• CF patients with A561E (and likely also those with Y1029X) can potentially benefit from lumacaftor.
• The methodology used here exemplifies how ex vivo approaches may apply personalized therapies to CF and possibly other respiratory diseases.

## 1 Introduction

Cystic fibrosis (CF), the most common life-shortening genetic disease affecting approximately 80,000 people worldwide (Bobadilla et al., 2002, The Molecular Genetic Epidemiology, na, Farrell, 2008, Rodrigues et al., 2009), is caused by mutations in the gene encoding the cystic fibrosis transmembrane conductance regulator (CFTR) protein. The dominating clinical CF phenotype is the respiratory disease, being hallmarks of this disease the very thick mucus obstructing the airways, chronic inflammation and persistent infections mostly by *Pseudomonas aeruginosa*, which altogether lead to eventual impairment of respiratory function (Bell et al., 2015). Other CF symptoms include pancreatic dysfunction, elevated sweat electrolytes and male infertility, but the progressive loss of lung function remains the leading cause of morbidity and mortality (Bell et al., 2015).

Most current treatments for CF target the secondary effects of dysfunction of CF lung disease to alleviate its symptoms (mucolytics, antibiotics, etc). However, new therapies modulating defective CFTR, the basic defect underlying CF, have started to hit the clinic and several others are in trial or in development.

CFTR is an essential epithelial anion channel that regulates several other channels and transporters, altogether regulating ion homeostasis and water content of epithelia surfaces. This member of the ABC transporter family has been reported to host > 1900 mutations, presumed to be CF-causing, albeit some still of unknown impact (Sosnay et al., 2013). Such genetic diversity makes the drug discovery based on protein rescue a huge task. Therefore, CFTR mutations are grouped into 6 functional classes, so as to apply the same CFTR-corrective therapy within each functional class to drastically downscale the drug discovery pipeline (for reviews see Bell et al., 2015, Amaral and Farinha, 2013). Notwithstanding, one single mutation — F508del, occurring in ~ 85% of CF patients in at least one allele and associated with severe CF —remains the most common CF mutation worldwide. F508del-CFTR is associated with defective traffic (Class II) which precludes it from reaching the cell surface [reviewed in (Amaral, 2004)].

The most attractive CFTR-modulator therapies involve: correctors to rescue F508del-CFTR to the cell surface and potentiators to restore CFTR mutants which exhibit a channel regulation defect (Class III). Potentiator ivacaftor, the first CFTR-targeting drug, was recently approved by FDA/EMA, albeit for a rare mutation — G551D (Ramsey et al., 2011) and for other Class III CFTR mutations (Van Goor et al., 2014, De Boeck et al., 2013), which, altogether only target ~ 5% of CF patients worldwide.

For CF patients with the most frequent mutation F508del, the best investigational drug is VX-809 (or lumacaftor, Vertex), reported to rescue ~ 25% CFTR activity in F508del/F508del primary human bronchial (HBE) cells (Van Goor et al., 2011). Very recently, this investigational drug, in combination with ivacaftor, succeeded in showing significant efficacy in a Phase III clinical trial on F508del/F508del patients (Prease release), an achievement that will likely result in its FDA-approval. lumacaftor, plausibly acting by correcting the folding of a critical contact site in CFTR structure (Farinha et al., 2013), rescues the abnormal intracellular localization of F508del-CFTR to the cell surface, a traffic defect that is common to all Class II CFTR mutants.

Our aim here was to assess efficacy of lumacaftor on other CFTR mutants with the same traffic defect as F508del (Class II (Amaral and Farinha, 2013)). Mutations tested here include: A561E, quite frequent in Southern-European and South-American countries like in Portugal (Mendes et al., 2003), Spain (Moya-Quiles et al., 2009) and Brazil (Servidoni et al., 2013) and N1303K, linked to ancient Mediterranean populations (Bobadilla et al., 2002). In addition we tested VX-809 in HBE cells bearing 2 nonsense mutations: G542X and Y1092X, both in heterozygosity with F508del.

Our data in primary HBE cells show that lumacaftor rescues A561E at equivalent efficacy of F508del, but N1303K is not significantly rescued. Data also show that VX-809 rescues F508del in cells from different donors with great variability. Compound C18 (lumacaftor analogue, also reported to rescue F508del) failed to rescue A561E-CFTR, thus plausibly rescuing CFTR by a different mechanism of action than lumacaftor.

We conclude that CF patients with the A561E mutation can potentially benefit from lumacaftor and personalized medicine is the way forward to tackle CF.

## 2 Methods

### 2.1 Culture Conditions of Primary Human Bronchial Epithelial Cells

Human lung tissues from CF donors with the F508del/F508del (2 donors), A561E/A561E, N1303K/G542X, F508del/G542X and F508del/Y1092X genotypes, were obtained from the Cardio-Thoracic Surgery Department (University Hospital la Fe, Valencia, Spain) after receiving patient's written consent and approval by the hospital Ethics Committee. Primary cultures of human bronchial epithelial (HBE) cells were isolated as described previously (Fulcher and Randell, 2013) and then expanded and grown on collagen IV-coated porous membranes (Snapwell, Corning-Costar®, Tewksbury, MA, USA) also as described (Moniz et al., 2013). Transepithelial electrical resistance (TEER) of the HBE monolayers growing on porous membranes was measured with the chopstick electrode (STX2 from WPI®, Berlin, Germany) and electrophysiological analyses were carried out in monolayers with resistance values above 600 Ω·cm^2^. No significant differences were measured for HBE monolayers with the different genotypes (Fig. S1). HBE cells were incubated with 3 μM VX-809 (lumacaftor), 5 μM C18, or DMSO vehicle alone (0.1%, v/v) for 24 h prior to the experiments as a control.

### 2.2 Micro-Ussing Chamber Recordings

Monolayers of HBE cells were mounted in micro-Ussing chambers and analysed under open-circuit conditions at 37 °C, as before (Moniz et al., 2013). Values for the transepithelial voltage V_te_ were referenced to the basal surface of the epithelium. Transepithelial resistance R_te_ was determined by applying 1 s current pulses of 0.5 μA (5 s-period). The cAMP-stimulated CFTR equivalent short-circuit currents (I_eq-sc_) were calculated according to Ohm's law from V_te_ and R_te_ (I_eq-sc_ = V_te _/ R_te_), with appropriate correction for fluid resistance. Ringer solution Cl^−^ concentrations apical and basal were 30 mM and 145 mM respectively and pH adjusted to 7.4. Following a 20-min equilibrium period, amiloride (20 μM) added to the luminal side to block epithelial Na^+^ channel (ENaC)-mediated Na^+^ flux, then cAMP agonist, 2 μM forskolin (Fsk), the CFTR potentiator 25 μM genistein (Gen), and the CFTR channel blocker CFTR Inh_172_ (30 μM) were added sequentially.

### 2.3 Statistical Analysis

Statistical comparisons were made using two-tailed Student's t tests and statistically significance was considered for p ≤ 0.05.

## 3 Results

### 3.1 Response to lumacaftor for Class II Mutants Assessed by CFTR-Mediated Chloride Secretion

**Fig. 1:**
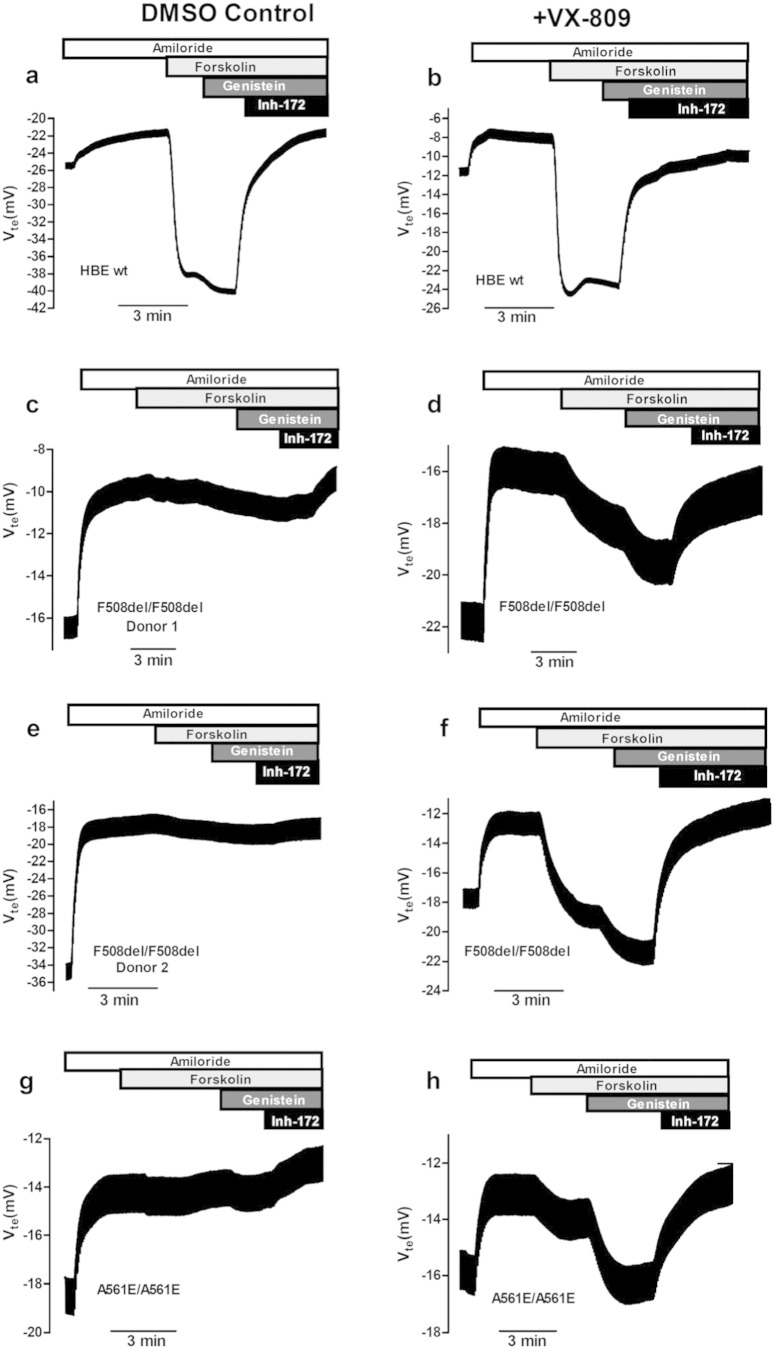
Effect of lumacaftor (VX-809) on cAMP-induced I_sc-eq_ in primary cultures of HBE cells from CF patients with class II mutations. Original Ussing chamber (open-circuit) recordings showing transepithelial voltage measurements (V_te_) obtained for CF primary airway HBE monolayers with different CFTR genotypes: wt/wt control (a, b); F508del/F508del-donor 1 (c, d); F508del/F508del-donor 2 (e, f); and A561E/A561E (g, h). Cells were preincubated for 24 h with either 3 μM/24 h lumacaftor/VX-809 (b, d, f, h) or DMSO (0.1%v/v) vehicle control (a, c, e, g). Amiloride (20 μM) was kept during the whole experiment duration to avoid interference of ENaC-mediated Na^+^ currents. Negative transepithelial voltage (V_te_) deflections are observed following the application of luminal forskolin alone (Fsk, 2 μM) or with genistein (Gen, 25 μM). The latter are fully reverted by application of 30 μM Inh_172_, a specific CFTR inhibitor (see also values in Table S1).

**Fig. 2:**
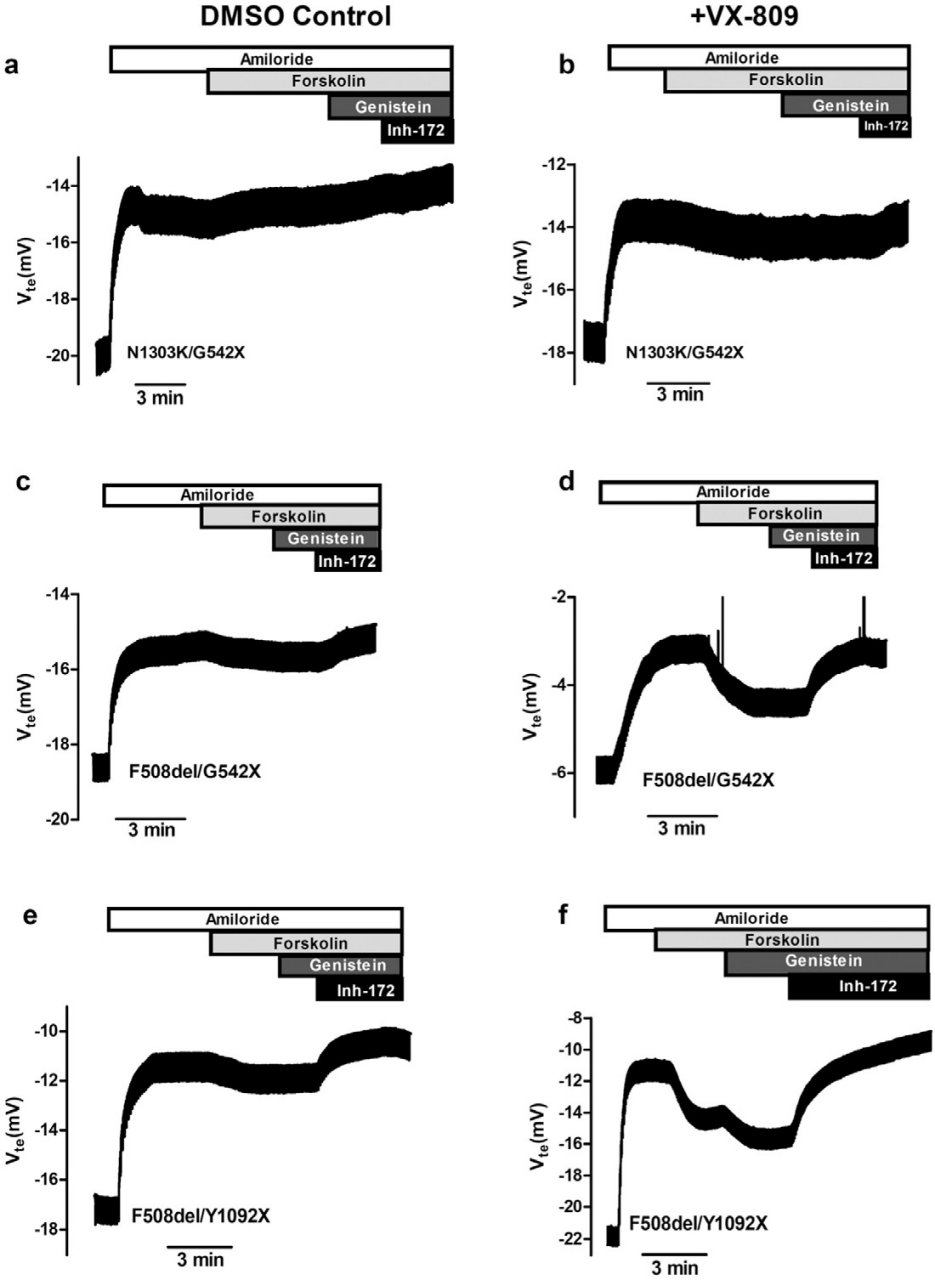
Effect of lumacaftor (VX-809) on cAMP-induced I_sc-eq_ in primary cultures of HBE cells from CF patients with different CFTR mutations. Original Ussing chamber (open-circuit) recordings showing transepithelial voltage measurements (V_te_) obtained for CF primary airway HBE monolayers with different genotypes: N1303K/G542X (a, b); F508del/G542X (c, d); and F508del/Y1092X (e, f). Cells were pre-incubated for 24 h with either 3 μM/24 h lumacaftor/VX-809 (b, d, f) or DMSO (0.1%v/v) vehicle control (a, c, e). Other conditions as described in Fig. 1 legend (see also values in Table S1).

The effects of 24 h-treatment with lumacaftor were assessed here by determining CFTR-mediated Cl^−^ secretion in HBE cells from CF donors with the following genotypes (Fig. 1): wt/wt control (a, b); F508del/F508del-Donor 1 (c, d); F508del/F508del-Donor 2 (e, f); A561E/A561E (Fig. 1g, h) and also on the additional genotypes (Fig. 2): N1303K/G542X (a, b), F508del/G542X (c, d); F508del/Y1092X (e, f). Since G542X is a “null” variant (i.e., generating no protein) results on the latter are representative of the N1303K variant, albeit in a single dose. The equivalent short-circuit current (I_eq-SC_) as a measurement of CFTR-mediated Cl^−^ secretion (see Methods) was determined for cAMP-stimulation by both Forskolin (I_eq-SC-Fsk_) alone or with Genistein (I_eq-SC-Fsk + Gen_).

**Fig. 3:**
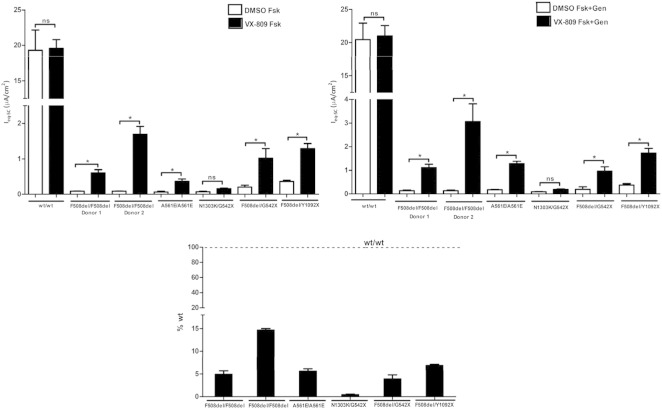
Summary of the effect of lumacaftor (VX-809) on HBE cells from CF patients with different genotypes. Graphs represent values of I_eq-sc_ (μA/cm^2^) calculated from voltage deflection obtained for the responses to Fsk (a) or to Gen + Fsk (b), after 24 h treatment with 0.1% DMSO (white bars) or 3 μM VX-809 (black bars) for HBE cells with different genotypes, as indicated below the graphs. (c) Percentage of I_eq-sc_ rescue in response to Forskolin plus Genistein (I_eq-sc-Fsk + Gen_) after VX-809 vs DMSO vs non-CF cells (see also Table S2). *indicates statistically significant (p > 0.05) and “ns” not significant.

These results show that Fsk + Gen responses of F508del/F508del (2 donors), A561E/A561E F508del/G542X and F508del/Y1092X cells after VX-809/lumacaftor treatment were significantly different from those under DMSO, while that of N1303K/G542X cells was not significantly different (Fig. 3b).

The respective Fsk responses after VX-809 (Fig. 3a) were lower that the corresponding Fsk + Gen responses, as expected due to the absence of the potentiator, but differences between VX-809 and DMSO-treated cells were similarly significant.

The effect of VX-809 was also estimated as fold-increase of equivalent short-circuit currents in response to Forskolin plus Genistein (I_eq-sc-Fsk + Gen_) after VX-809 vs DMSO (Table S2) and as percentage of rescue vs non-CF cells (Fig. 3c, Table S2). These data again clearly show a positive effect of VX-809 on HBE cells with genotypes F508del/F508del (both donors), A561E/A561E, F508del/G542X and F508del/Y1092X but not on N1303K/G542X cells. Of note is the striking difference between the responses of the two F508del/F508del donors.

It is also interesting to note the difference in responses by the F508del/G542X and F508del/Y1092X cells. Since F508del/Y1092X cells (but not F508del/G542X cells) already exhibit levels of I_eq-sc-Fsk_ or I_eq-sc-Fsk + Gen_ before VX-809, we assessed the levels of the non-F508del transcripts in these two cells, i.e., those with the stop mutation to determine the respective levels of nonsense-mediated mRNA decay (Table S3). Data, show that the levels of Y1092X-transcripts are higher than those from G542X (Table S3), indicating that Y1092X transcripts are less prone to degradation through nonsense-mediated decay.

### 3.2 Response of A561E/A561E HBE Cells to lumacaftor and Compound C18

**Fig. 4:**
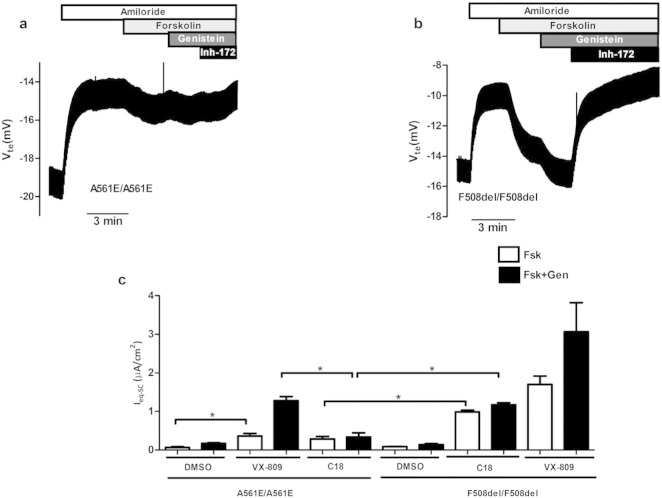
Original tracings and summary of the effect of C18 in A561E/A561E and F508del/F508del primary HBE cells. (a, b) represent original Ussing chamber (open-circuit) recordings obtained for the analysis of CF primary airway HBE monolayers with A561E/A561E and F508del/F508del treated with 5 μM C18 for 24 h. (c) Graph represents summary of I_sc-eq_ (μA/cm^2^) values obtained for responses to Fsk (white bars) or Fsk + Gen (black bars) after 24 h-treatment with DMSO, VX-809/lumacaftor or C18 as indicated (see also Table S4). * indicates statistically significant (p > 0.05).

A561E/A561E HBE cells were also treated with C18 compound, described as a lumacaftor analogue (Eckford et al., 2014). As demonstrated by the original tracing in Fig. 4a, the responses elicited by either Fsk or Fsk + Gen in A561E/A561E cells pre-incubated with C18 are lower than those in F508del/F508del cells (Fig. 4b) and this difference is statistically different (Fig. 4c). Moreover, the response of A561E/A561E cells after C18 treatment is also significantly lower than that in lumacaftor-treated cells, while those of F508del/F508del cells after C18 and VX-809 are similar (Fig. 1c, Table S4). Indeed, the Fold rescue of I_eq-sc-Fsk + Gen_ in A561E/A561E cells after C18 treatment was 1.93 ×, while this value was 6.51 × F508del/F508del cells. Similarly, the percentages of rescue by C18 vs non-CF cells (wt/wt) were ~ 0.8% and ~ 5.0% for A561E/A561E F508del/F508del cells, respectively.

These data also indicate that the response of A561/A561E HBE cells to C18 is lower than to lumacaftor, when these cells are stimulated by Gen, but interestingly, not when stimulated only by Fsk. To confirm these data, Western blot was performed in BHK cells stably expressing F508del or A561E mutant protein. Data show that VX-809 rescues both F508del and A561E-CFTR, while C18 failed to rescue A561E-CFTR but not in F508del-CFTR protein (Fig. S2). These data are thus consistent with those obtained for A561E/A561E cells treated with C18.

## 4 Discussion

CF has been for a long time a paradigmatic monogenic disease for the advancement of both biomedical science and clinical practice. CF also pioneers drug discovery programmes for rare diseases, as recently demonstrated by the recent approval for the clinic of ivacaftor, a compound that treats the basic gating defect associated with Class III CFTR protein mutants.

However, this novel treatment only applies to 9 of the 1900 CFTR gene mutations reported to date (~ 5% of all CF patients). The CF community should thus work fast to determine whether ivacaftor, or the investigational drug lumacaftor (for Class II mutants) rescue other CFTR mutants and thus can be extended to more CF patients.

The objective of this study was to evaluate the effect of lumacaftor on additional CFTR mutants which, similarly to the most frequent mutation F508del (Van Goor et al., 2011), also affect the traffic of the protein to the plasma membrane. To this end, we used the best known in vitro CF model of human airways, consisting in primary cultures of human bronchial epithelial cells (HBE) grown as monolayers in porous filters and we used then for CFTR bioelectric measurements in perfused micro-Ussing chambers (Moniz et al., 2013).

Our data show that the effect of lumacaftor on A561E/A561E HBE cells was equivalent to that of this investigational drug in F508del/F508del cells. Indeed, after the incubation of A561E/A561E cells with 3 μM lumacaftor for 24 h, responses obtained in the Ussing chamber were 7-fold higher than when cells were incubated with DMSO-vehicle, representing ~ 6% of rescue vs non-CF cells. For F508del/F508del cells responses of lumacaftor-treated cells were 8/14-fold higher than those under DMSO, representing 5–15% of rescue vs non-CF cells. These data seem to indicate that the previously characterized trafficking defect of the A561E-CFTR protein (Mendes et al., 2003) can be, as least partially, corrected by lumacaftor. Interestingly, a previous study showed that A561E-CFTR can be rescued to the cell surface by the same genetic revertants as F508del-CFTR (Roxo-Rosa et al., 2006). In another more recent study, the A561E-CFTR channel was also described to have similar mechanisms of dysfunction and response to potentiators as F508del-CFTR (Wang et al., 2014). Of note is the striking difference between the responses of the two F508del/F508del donors, which can potentially be a predictor of variable patients' response to this investigational drug. Data presented here also show a positive effect of VX-809 on HBE cells with genotypes F508del/G542X (~ 4% vs non-CF) and F508del/Y1092X (~ 7% vs non-CF) but not on N1303K/G542X cells.

Our data also lead to the conclusion that the A561E responses to lumacaftor and its analogue C18 do not totally overlap, as observed from the significantly lower Fsk + Gen response of A561E/A561E cells pre-incubated with C18 vs those under lumacaftor. In contrast, F508del-CFTR responds similarly to both correctors, similarly to what was previously reported (Eckford et al., 2014). Noticeably, however, the Fsk-response of C18-treated A561E/A561E cells is significantly higher than in the DMSO-treated cells (Fig. 4c). Therefore, the failure in C18-treated to significant respond to the further stimulation by potentiator Genistein, might be due to a possible dual activity (corrector and potentiator) of the C18 compound as suggested (Eckford et al., 2014), which likely would be overlapping with that of Gen. Nonetheless, C18 also failed to rescue A561E-CFTR as assessed by Western blot, while VX-809 induces a detectable levels of mature A561E-CFTR (Fig. S2). Although those authors have used a higher C18 concentration for a longer pre-incubation time (6 μM/48 h) (Eckford et al., 2014), the conditions we employed here (5 μM/24 h) were also used in another study (Holleran et al., 2012) and in fact correspond to the concentration range recommended by CFFT (3–6 μM).

In contrast to the effect on A561E/A561E HBE cells, the magnitude of the response of lumacaftor-treated N1303K/G542X cells was just slightly higher by ~ 2-fold (both under Fsk and Gen) and not statistically different from that in DMSO-treated cells. Moreover, the percentage of rescue vs non-CF cells was barely 0.5%, thus showing a lack of an effect by VX-809 on N1303K. Two hypotheses may account for this lack of a significant response. Firstly, N1303K located in the second nucleotide binding domain (NBD2) of CFTR protein, may cause a different structural defect from that of F508del or A561E, both located in NBD1. Indeed, recent studies have suggested that the putative binding site of VX-809/lumacaftor is a “structural pocket” between NBD1 and the fourth intracellular loop (ICL4) of the second transmembrane domain (Farinha et al., 2013, He et al., 2013). Plausibly, NBD2-located N1303K creates a distinct defect which unlikely would be corrected by the lumacaftor. Secondly, it is possible that the response of a single copy of N1303K (the other CFTR allele is G542X, a “null” variant) may be insufficient to observe an effect similar in magnitude to that of A561E/A561E or F508del/F508del cells. Contradicting the latter hypothesis are the positive responses of the F508del/G542X and F508del/Y1092X cells, showing that VX-809 can elicit a detectable effect on a single dose of F508del, in contrast to N1303K.

Interestingly, the response of F508del/Y1092X cells is almost double to that of F508del/G542X. While difference could be due to intrinsic responses of the F508del alleles from each of these donors, it is also plausible that the Y1092X mutation, given its localization towards the C-terminus of the protein, does not totally abolish the production of functional CFTR protein, in contrast to G542X. Indeed, these HBE cells already exhibit levels of I_eq-sc-Fsk_ or I_eq-sc-Fsk + Gen_ before VX-809, which are higher than those of F508del/F508del cells, suggesting that Y1092X-CFTR protein may elicit such response. Moreover, the levels of Y1092X-transcripts (Table S3) are higher than those from G542X, again indicating that Y1092X transcripts are less prone to degradation through nonsense-mediated decay. It is thus likely that Y1092X originates CFTR protein with residual function with some positive response to VX-809.

In conclusion, our data suggest that CF patients bearing the A561E mutation, which is associated with a severe clinical phenotype and quite common in some countries (Mendes et al., 2003), can potentially benefit from lumacaftor treatment. Similarly, lumacaftor seems to have a positive impact on Y1092X. Our data also show that cells with only one copy of F508del-CFTR respond less to VX-809. Moreover, there is great variability in lumacaftor responses among F508del-homozygous cells from different donors. Importantly, the methodology used in this study exemplifies how ex vivo approaches may apply personalized therapies to Cystic Fibrosis and possibly other respiratory diseases. These data actually demonstrates the main topic of this study which is each patient should be tested individually for the responsiveness to the compounds.

## Conflicts of Interest

MDA has served as a consultant to Vertex and Galapagos, and has been supported to attend and to speak at Symposia (Novartis, Gilead and Vertex) and to participate in an educational grant programme by Facilitate Ltd. AS has served as a consultant to Vertex.

## Authors' Contributions

• Nikhil T Awatade — generated data, analysed data, wrote and revised manuscript
• Inna Uliyakina — generated data, analysed data, revised manuscript
• Carlos M Farinha — generated data, revised manuscript
• Luka A Clarke — generated data, revised manuscript
• Karina Mendes — generated data, revised manuscript
• Amparo Solé — collected patients samples, revised manuscript
• Juan Pastor — collected patients samples, revised manuscript
• Maria Margarida Ramos — generated data, analysed data, revised manuscript
• Margarida D. Amaral (guarantor of the paper) — designed experiments, analysed data, wrote manuscript, raised funding
