# Supplementary material for: Measurements of Functional Responses in Human Primary Lung Cells as a Basis for Personalized Therapy for Cystic Fibrosis
Source: eBioMedicine. 2014 Dec 17;2(2):147–53. doi: 10.1016/j.ebiom.2014.12.005 (PMC4484512; doi:10.1016/j.ebiom.2014.12.005)
Supplement: Supplementary file 1 — Supplementary material. [file mmc1.docx]

**Supplementary Data to Awatade et al "Measurements of Functional Responses in Human Primary Lung Cells as a Basis for Personalised Therapy for Cystic Fibrosis"**

**Supplementary Figure 1**

**Figure S1.** Transepithelial electrical resistance of the HBE cell monolayers. Transepithelial resistance (TER) was measured for all different HBE cultures grown under various conditions as indicated. No significant differences were found among TER values for the different cultures.

**Supplementary Table 1**

**Table S1. Data for the equivalent short-circuit current in HBE cells from CF patients with different genotypes.** Data (2 top rows) represent values of equivalent short-circuit currents (I_eq-SC_) determined for HBE cells from CF patients with different genotypes as indicated under stimulation by Forskolin alone (Fsk) or also Genistein (Fsk+Gen) respectively, after DMSO or VX-809 (3µM/24h) treatments. Data on the effect of CFTR-specific inhibitor to I_eq-SC-_ _Fsk+Gen_. are also shown (3^rd^ row). Estimated responses to VX-809 are expressed as the variation in equivalent short-circuit currents (ΔI_eq-sc_) to Fsk alone or Fsk+Gen (2 bottom rows). Values are represented as (mean ±SEM µA/cm2); (n) indicates number of experiments.

|  | **wt/wt** | | **F508del/F508del**  **(Donor 1)** | | **F508del/F508del**  **(Donor 2)** | | **A561E/A561E** | | **N1303K/G542X** | | **F508del/G542X** | | **F508del/Y1092X** | |
| --- | --- | --- | --- | --- | --- | --- | --- | --- | --- | --- | --- | --- | --- | --- |
|  | **DMSO**  **(n=6)** | **VX 809**  **(n=6)** | **DMSO**  **(n=3)** | **VX 809**  **(n=3)** | **DMSO**  **(n=3)** | **VX 809**  **(n=3)** | **DMSO**  **(n=3)** | **VX 809**  **(n=3)** | **DMSO**  **(n=3)** | **VX 809**  **(n=5)** | **DMSO**  **(n=5)** | **VX 809**  **(n=5)** | **DMSO**  **(n=5)** | **VX 809**  **(n=6)** |
| **I_sc-eq-Fsk_** | 19.30±2.855 | 19.56±1.257 | 0.088±0.007 | 0.605±0.087 | 0.096±0.057 | 1.696±0.217 | 0.065±0.021 | 0.364±0.067 | 0.073±0.015 | 0.143±0.029 | 0.204±0.056 | 1.022±0.266 | 0.361±0.036 | 1.291±0.143 |
| **I_sc-eq-Fsk+Gen_** | 20.46±2.486 | 21.01±1.583 | 0.138±0.028 | 1.108±0.15 | 0.180±0.074 | 3.063±0.753 | 0.174±0.014 | 1.283±-0.103 | 0.096±0.005 | 0.188±0.023 | 0.195±0.110 | 0.969±0.178 | 0.380±0.055 | 1.727±0.199 |
| **I_sc-eq-Inh172Fsk+Gen_** | 0.784±0.231 | 2.946±0.597 | -0.340±0.134 | -1.544±0.152 | -380±0.07 | -3.499±0.870 | -0.497±0.081 | -1.427±0.119 | -0.156±0.045 | -0.346±0.084 | -0.457±0.202 | -1.034±0.206 | -0.727±0.062 | -2.063±0.248 |
| **ΔI_sc-eq-Fsk_**  **(VX 809-DMSO)** | - | 0.260±2.56 | - | 0.516±0.087 | - | 1.600±0.058 | - | 0.299±0.07 | - | 0.0704±0.029 | - | 0.818±0.26 | - | 0.9302±0036 |
| **ΔI_sc- Fsk+Gen_**  **(VX 809-DMSO)** | - | 0.550±1.91 | - | 0.970±0.16 | - | 2.883±0.075 | - | 1.109±0.103 | - | 0.092±0.023 | - | 0.775±0.178 | - | 1.347±0.056 |

**Supplementary Table 2**

**Table S2** – **Fold increase of equivalent short-circuit currents in response to Forskolin plus Genistein (Ieq-sc-Fsk+Gen) after VX-809 vs DMSO and percentage of rescue vs non-CF cells (wt/wt).**

| **HBE Cell Genotype** | **ΔI_eq-SC_ fold-increases after VX-809 vs DMSO** | **% rescue vs wt/wt** |
| --- | --- | --- |
| **F508del/F508del (Donor 1)** | 8.03x | 4.92±0.78% |
| **F508del/F508del (Donor 2)** | 17.01x | 14.65±0.37% |
| **A561E/A561E** | 7.37x | 5.63±0.50% |
| **N1303K/G542X** | 1.96x | 0.46±0.11% |
| **F508del/G542X** | 4.96x | 3.93±0.87% |
| **F508del/Y1092X** | 4.54x | 6.84±0.27% |

**Supplementary Table 3**

**Table S3 - QPCR data of the relative abundance of CFTR alleles**

|  | **Fold Change (non-F508del/F508del) allele abundance** | | |
| --- | --- | --- | --- |
|  | **Replicate 1** | **Replicate 2** | **Mean** |
| F508del/Y1092X  (+VX-809) | 0.38 | 0.41 | 0.40 |
| F508del/Y1092X | 0.37 | 0.33 | 0.35 |
| F508del/G542X | 0.29 | 0.26 | 0.27 |

QPCR data showing relative abundance of CFTR alleles from the Human bronchial epithelial cells with F508del/Y1092X and F508del/G542X muatations

The RNA was extracted from HBE cells with F508del/Y1092X mutation (VX-809 3µM/ 24h) previously used in Ussing chambers measurements and cDNA was synthesised by RT-PCR. In a next step cDNA together with more two cDNA corresponding to the genotypes F508del/Y1092X and F508del/G542X (RNA extracted from bronchi samples) were used in a qPCR experiment. Considering the Y1092X and G542X as "non-F508del alleles", we compare their expression levels vs those of the F508del allele. Although, the allele Y1092X +VX-809 (top row) presents slightly higher expression levels when comparing with the levels of this allele without VX-809 (middle row) the difference is not significant. According to the results of fold change (non-F508del/F508del allele abundance), we can conclude that the allele G542X is associated with lower levels of transcripts, i.e., higher levels of mRNA degradation (likely via nonsense-mediated decay), thus leading to less production of protein.

**Supplementary Table 4**

**Table S4** – **Effect of C18 in A561E/A561E and F508del/F508del cells**

|  | **F508del/F508del** | **A561E/A561E** |
| --- | --- | --- |
| **I_eq-sc-_Fsk** | 0.987±0.043 | 0.287±0.06 |
| **I_eq-sc-_Fsk+Gen** | 1.173±0.052 | 0.336±0.110 |
| **I_eq-sc-_Fsk+Gen+Inh172** | -1.473±0.126 | -0.754±0.136 |
| **Δ I_eq-sc-_Fsk** | 0.891±0.055 | 0.222±0.071 |
| **Δ I_eq-sc-_Fsk+Gen** | 0.993 ±0.075 | 0.162±0.110 |
| **Fold rescue of I_eq-sc-_Fsk+Gen** | 6.51x | 1.93x |
| **% to wt** | 5.04±0.36% | 0.82±0.53% |

*DMSO values as in Table S1

**Supplementary Figure 2**

Western blot quantification in BHK cells expressing F508del or A561E mutant protein. Effects of C18 and VX-809 was assessed in F508del and A561E-CFTR expressed in BHK cells.


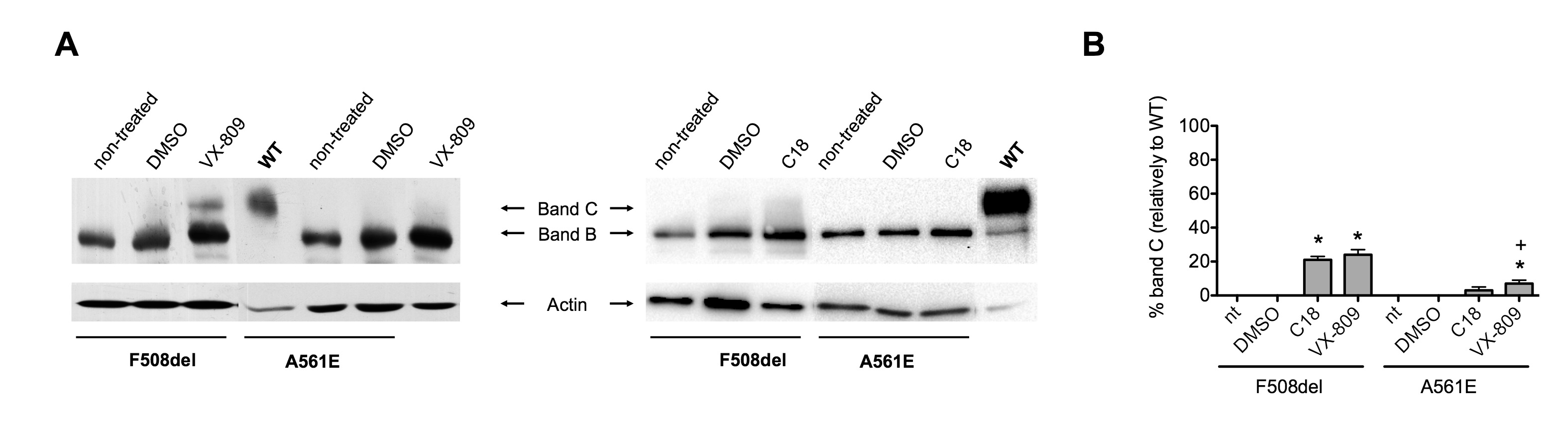


**Figure S2. Effect of C18 and VX-809 on rescuing of F508del- and A561E in stable BHK cells**. F508del-CFTR to a level that’s not different from each other. VX-809 rescues also A561E-CFTR, but C18 failed to rescue mutant CFTR protein, *means statistically different (p<10^-9^) from DMSO, +means statistically different from C18. These data support the Ussing chamber data from figure 4 of the manuscript.
